# Supplementary material for: Splice-Junction-Based Mapping of Alternative Isoforms in the Human Proteome
Source: Cell Rep. Author manuscript; Available in PMC 2020 Jan 15. (PMC6961840; doi:10.1016/j.celrep.2019.11.026)
Supplement: 2 [file NIHMS1546469-supplement-2.zip › DF1/PXD006675/VentricularSeptum/VentricularSeptum_2_MYBPC3_RTDSHEDTGILDFSSLLK.pdf]

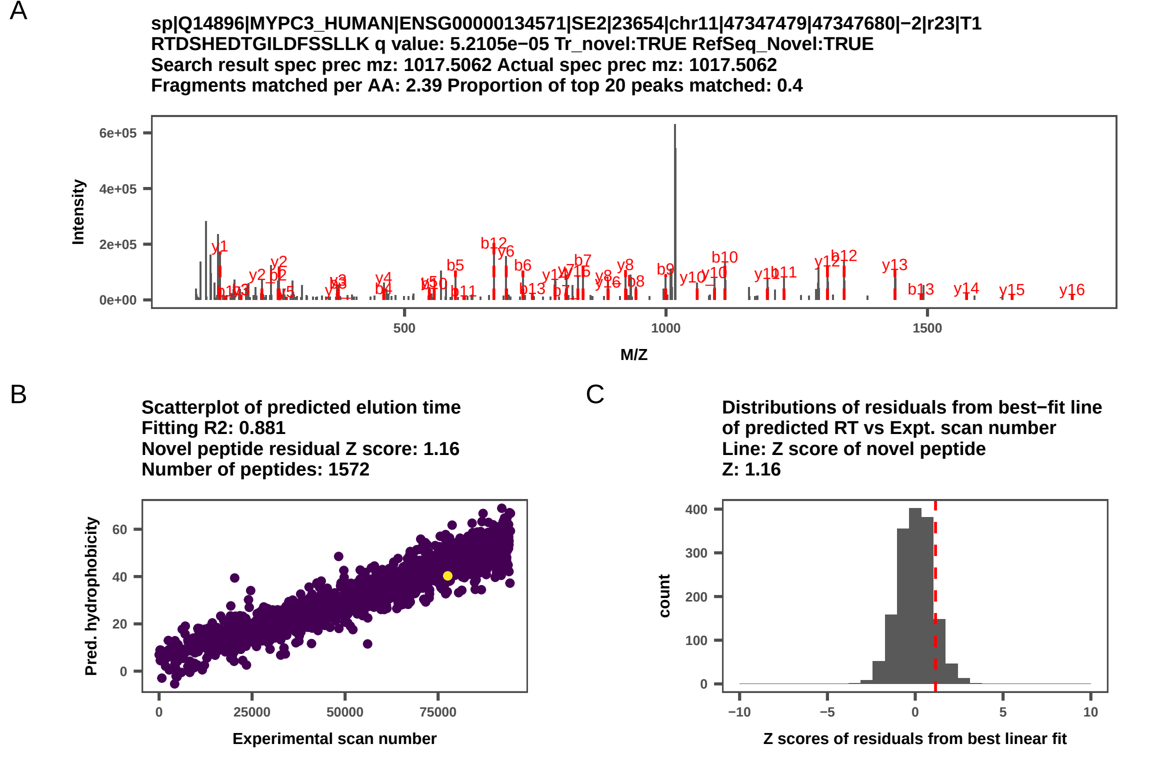
A
sp|Q14896|MYPC3_HUMAN|ENSG00000134571|SE2|23654|chr11|47347479|47347680|−2|r23|T1
RTDSHEDTGILDFSSLLK q value: 5.2105e−05 Tr_novel:TRUE RefSeq_Novel:TRUE
Search result spec prec mz: 1017.5062 Actual spec prec mz: 1017.5062
Fragments matched per AA: 2.39 Proportion of top 20 peaks matched: 0.4
Intensity
6e+05
4e+05
2e+05
0e+00
500
1000
1500
M/Z
B
Scatterplot of predicted elution time
Fitting R2: 0.881
Novel peptide residual Z score: 1.16
Number of peptides: 1572
Pred. hydrophobicity
60
40
20
0
0
25000
50000
75000
Experimental scan number
C
Distributions of residuals from best−fit line
of predicted RT vs Expt. scan number
Line: Z score of novel peptide
Z: 1.16
count
400
300
200
100
0
−10
−5
0
5
10
Z scores of residuals from best linear fit
